# Dynamics of detailed components of metabolic syndrome associated with the risk of cardiovascular disease and death

**DOI:** 10.1038/s41598-021-83118-y

**Published:** 2021-02-11

**Authors:** Ting-Yu Lin, Kuo-Liong Chien, Yueh-Hsia Chiu, Pi-Chun Chuang, Ming-Fang Yen, Hsiu-Hsi Chen

**Affiliations:** 1 Institute of Epidemiology and Preventive Medicine, College of Public Health, National Taiwan University, Room 533, No. 17, Hsuchow Road, Taipei, 100 Taiwan; 2 Department of Internal Medicine, National Taiwan University Hospital, Taipei, Taiwan; 3 Department of Health Care Management, College of Management, Chang Gung University, Taoyuan, Taiwan; 4 Division of Hepatogastroenterology, Department of Internal Medicine, Kaohsiung Chang Gung Memorial Hospital, Kaohsiung, Taiwan; 5 School of Medicine, College of Medicine, Fu Jen Catholic University, New Taipei, Taiwan; 6 Taiwan Medical Education Department, Far Eastern Memory Hospital, New Taipei, Taiwan; 7 School of Oral Hygiene, College of Oral Medicine, Taipei Medical University, Taipei, Taiwan

**Keywords:** Cardiology, Diseases, Medical research

## Abstract

Few studies quantify a cascade of dynamic transitions on the detailed components of metabolic syndrome (MetS) and subsequent progressions to cardiovascular disease (CVD) and its death. A total of 47,495 subjects repeatedly attending a community-based integrated screening program in Taiwan were recruited. The refined MetS-related classification (RMRC) in relation to five criteria of MetS was defined as free of metabolic disorder (FMD, none of any criteria), mild metabolic disorder (MMD, 1–2 criteria) and MetS. A multistate Markov model was used for modelling such a multistate process. The estimated progression rate from FMD to MMD was 44.82% (95% CI 42.95–46.70%) whereas the regression rate was estimated as 29.11% (95% CI 27.77–30.45%). The progression rate from MMD to MetS was estimated as 6.15% (95% CI 5.89–6.42%). The estimated annual incidence rates of CVD increased with the severity of RMRC, being 1.62% (95% CI 1.46–1.79%) for FMD, 4.74% (95% CI 4.52–4.96%) for MMD, to 20.22% (95% CI 19.52–20.92%) for MetS. The estimated hazard rate of CVD death was 6.1 (95% CI 4.6–7.7) per thousand. Elucidating the dynamics of MetS-related transition and quantifying the incidence and prognosis of CVD provide a new insight into the design and the evaluation of intervention programs for CVD.

## Introduction

Metabolic syndrome (MetS) is a complex disorder defined by a cluster of interrelated factors associated with an increased risk for coronary heart disease (CHD), cardiovascular atherosclerotic diseases (CVD), and type 2 diabetes mellitus^1^. Although the underlying pathologic mechanisms are obscure^2^, the syndrome still attracts much attention due to the prevailing MetS in parallel with the epidemic of CVD worldwide^3^. The current evidence indicates that around 20–30% of the adult population can be classified as MetS in most countries^4^. A meta-analysis study, involving a total of 951,083 subjects, revealed that individuals afflicted with MetS have at least a 1.5-fold increase in all-cause mortality rates and a twofold increased risk of CVD^5,6^. Being aware of the epidemic condition and the impact of the MetS on CVD and CVD-specific mortality in the general population plays a crucial role in forming public health policies and clinical guidelines for its prevention and treatment.

Two unique characteristics of MetS can be specified including the progressive property of long natural course in the absence of appropriate intervention and treatment and heterogeneity resulting from the clustering of a constellation of risk factors on cardiovascular and its related chronic diseases. From the viewpoint of public health, both may affect not only the incidence rate of disease but also the duration of disease staying in chronic state. Despite a number of studies on MetS, the majority of studies have focused on the classification of MetS into two dichotomous states, MetS and non-MetS and very few studies put emphasis on the multistep progressive property of the natural history by providing the refined MetS-related classification (RMRC), consisting of no factor (free of metabolic disorder, FMD), one factor or two factors (mild metabolic disorder, MMD), MetS given the definition of having at least three criteria out of five factors (obesity, blood pressure, blood sugar, and lipid profiles). Elucidating such a complex pathophysiology in relation to the detailed components of MetS would make a contribution to providing a new insight into the prevention of future cardiovascular disease through the alteration of the individual components of MetS that are framed into the underlying multi-step disease process. To this end, a multistate MetS-related Markov process is used to study the dynamic changes between FMD, MMD, and MetS associated with the prognosis of two outcomes, CVD and its subsequent death from CVD, making allowance for other causes of death (OCD). Different epidemiological profiles on the dynamics of the multistate process provides a new insight into the classification of different risk groups for developing the precision intervention strategy in the future.

In this study, we aimed to construct a multistate Markov model to model age- and sex-specific dynamic changes of MetS status defined by the numbers of individual components and also the transition rates of each status leading to incidence and mortality of CVD taking OCD into account.

## Materials and methods

### Study cohort

**Figure 1:**
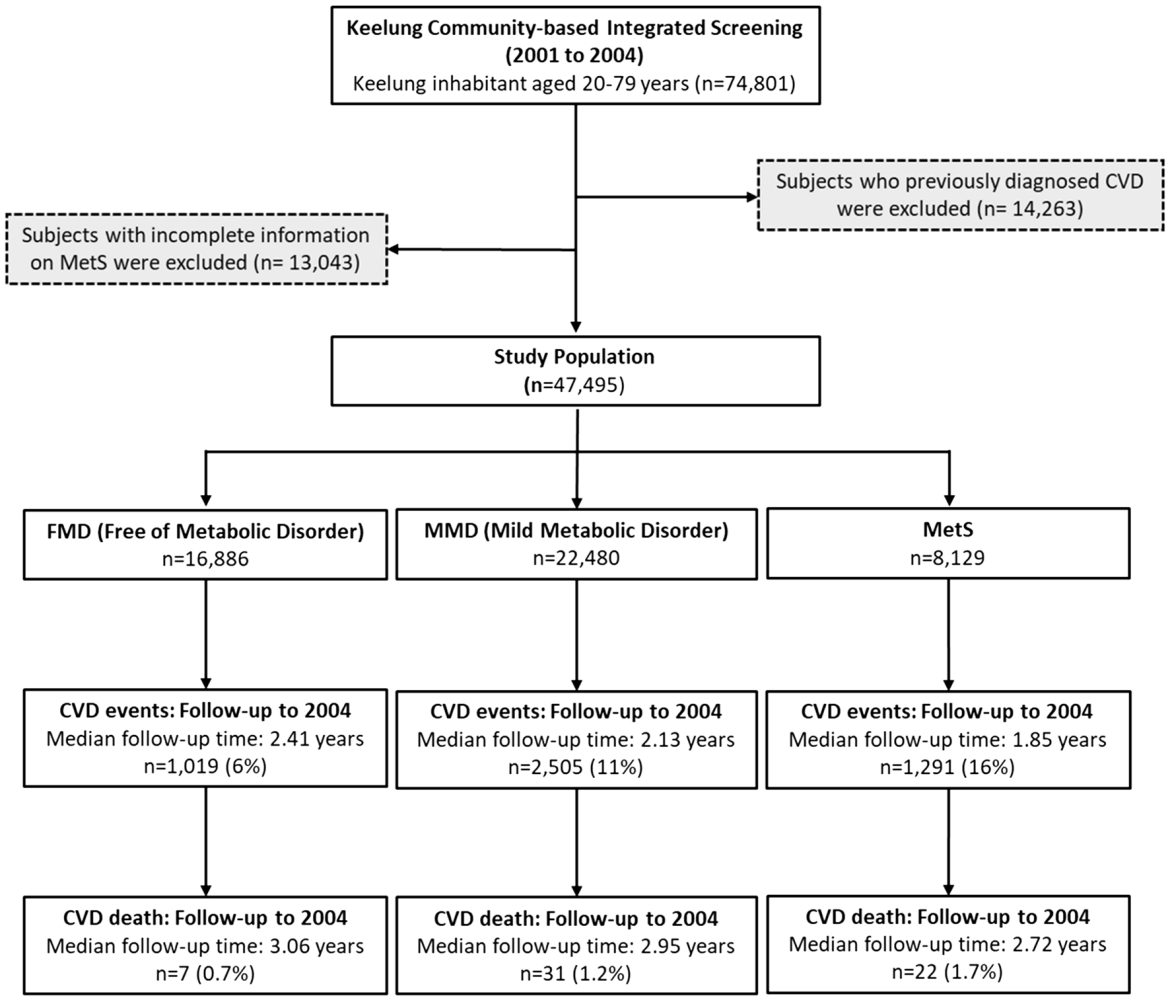
Flow chart of Study population.

The study cohort was derived from subjects attending the Keelung Community-based Integrated Screening (KCIS) project that is a multiple disease screening program and has been carried out in Keelung city since 1999. Details of the study design, implementation, follow up and the results of the KCIS program have been described in full elsewhere^7–9^. Briefly, a total of 74,801 residents aged 20–79 years old (including 46,459 women and 28,342 men) were enrolled in the KCIS program and attended screening for metabolic syndrome at least once between January 2001 and December 2004. After the exclusion of a further 14,263 subjects previously diagnosed CVD at baseline and 13,043 subjects with incomplete data for metabolic syndrome, the final effective samples on which the present analyses were based consisted of 47,495 subjects. In principle, annual screenings for metabolic syndrome are delivered to the eligible population. However, participants may re-attend the screen with irregular inter-screening intervals varying from 1 to 4 years. Figure 1 shows the recruitment and follow-up of study cohort. This study was approved by Research Ethics Committee of National Taiwan University Hospital (Registration number #201802004RIND). All procedures were performed in accordance with relevant guidelines and regulations and adhered to the tenets of the Declaration of Helsinki. Written informed consent of each participant was obtained at recruitment in the program.

### Data collection

Biochemical and anthropometric variables for defining MetS were collected through on-site screening for each participant in the KCIS program. A venous blood sample was taken after 8–12-h fasting for measuring plasma glucose, triglycerides, HDL-cholesterol, LDL-cholesterol and total cholesterol. Height, weight (measured to the nearest 0.1 kg), waist circumference (measured to nearest 0.1 cm) and other anthropometric factors were measured by the trained staffs. The same staff measured blood pressure in the right arm using an appropriately sized cuff and a standard mercury sphygmomanometer. Information on demographic characteristics (age and sex), life style factors related to MetS such as behaviors of smoking, alcohol drinking and physical activity, and personal disease history (diabetes mellitus, hypertension, cardiovascular and cerebrovascular disease, hyperlipidemia and stroke) for participants in the KCIS program was collected through face-to-face interview using a structured questionnaire administered by public health nurses. After screening, those with abnormal laboratory results would be referred to primary care clinics or hospitals for confirmatory clinical diagnosis.

### Definition of metabolic syndrome

The definition of MetS was defined in light of the joint scientific statement criteria with the requirement of more than three of the following five criteria^10^: (1) central obesity (waist circumference ≥ 80 cm for women, and ≥ 90 cm for men), (2) hypertriglyceride (triglyceride ≥ 150 mg/dL), (3) a low level of high density lipoprotein cholesterol (HDL-cholesterol) (HDL-cholesterol < 50 mg/dL for women and < 40 mg/dL for men), (4) an elevated blood pressure (systolic ≥ 130 mmHg or diastolic ≥ 85 mmHg), and (5) and hyperglycemia (fasting glucose ≥ 100 mg/dL). The cutoff of central obesity is adjusted for waist size in light of Asian subjects (WHO, 2001). Also note that subjects with personal history of hypertension, diabetes and hyperlipidemia under treatment were classified as the category of an elevated blood pressure, hyperglycemia and hyperlipidemia respectively.

### Refined MetS-related classification (RMRC)

For a better understanding of the dynamics of the natural course on the detailed components of MetS defined by five criteria as above, we provide the refined classification for those less than three individual components. Those with all items in the normal range are defined as free of metabolic disorder (FMD). Subjects meeting with 1–2 criteria are classified into mild metabolic disorder (MMD). Totally, there are three metabolic states defined by five criteria, FMD, MMD, and MetS (Refined MetS-related Classification, RMRC) that are used for the delineation of the following dynamics of three states and the further development of two prognostic outcomes, CVD and its death.

### Quantitative assessment of two prognosis outcomes

The two prognostics outcomes, CVD and its death, and OCD were obtained by linking the study cohort with Taiwan National Health Insurance Database and Taiwan National Mortality Registry to ascertain. The definition of CVD case in our study includes hypertensive heart disease (ICD code: 402); ischemic heart disease (ICD code 410–414); cardiomyopathy (ICD code: 425); arrhythmia (ICD code: 426–427); congestive heart failure (ICD code: 428); cerebrovascular disease (ICD code: 430–438); coronary artery bypass grafting; percutaneous transluminal angioplasty; diseases of arteries, arterioles, and capillaries (ICD code: 440–448). The proportions of CVD case were 6%, 11% and 16% after follow-up for FMD, MMD, and MetS, respectively. The corresponding average follow-up times were 2.41 ± 1.12 years, 2.13 ± 1.17 years, and 1.85 ± 1.15 years for FMD , MMD and MetS, respectively. The proportions of CVD-specific death were 0.7%, 1.2% and 1.7% and the corresponding average follow-up times were 3.06 ± 0.78 years, 2.95 ± 0.85 years, and 2.72 ± 0.94 years for FMD, MMD and MetS, respectively (Fig. 1).

### Statistical analysis

**Figure 2:**
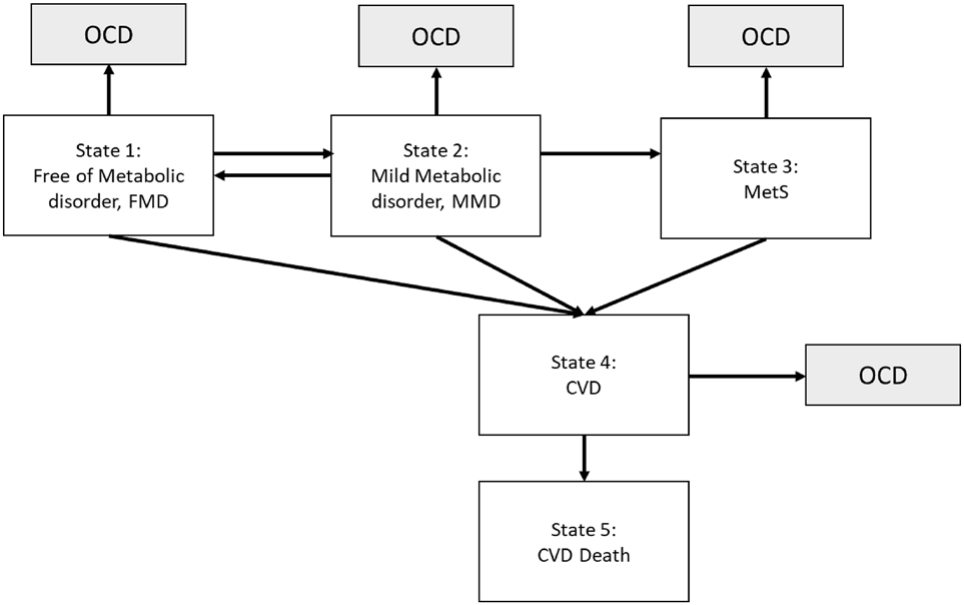
Six-state continuous-time Markov model.

Following Fig. 1, we used a six-state Markov process to describe the dynamics of natural course on RMRC associated with the risk for two prognostic outcomes, CVD case, and its death, making allowance for OCD as illustrated in Fig. 2. Such a multi-state Markov process has been widely applied to cancer and chronic diseases previously^11–13^. The six states in this study included three states of RMRC (FMD, MMD, and MetS) as defined above, two prognostic states CVD and its death, and OCD. We estimated the transition rates including the progression for the disease statuses moving toward MetS and subsequent progression to two prognostic outcomes and the regression from MMD to FMD. Note that we assume that the entry into the MetS state can only pass through MMD, but not directly from FMD. We allowed the possible regression from MMD to FMD but not allowed for MetS to MMD as we are interested in the natural course without being affected by interventions or therapeutic components. Once a patient was diagnosed as having metabolic syndrome, more aggressive interventions would be administered either through life style modifications or pharmacological treatments, which may interrupt the natural course although the regression from MetS to MMD is still observed bit such a circumstance is not a reflection of natural course of regression. Because we want to model the natural course of RMRC before developing into CVD, the regression from MetS to MMD was not allowed in the model. The details of six-state Markov model are elaborated in “Appendix”.

We applied the proportional hazard regression forms to quantify age- and six-specific transition rates between RMRC, age- and sex-specific incidence of CVD, and also age- and sex-specific death rate of CVD among CVD patients allowing for OCD. Modelling age and sex is entirely on the grounds of imbalanced distributions of age and sex. The estimates and 95% confidence intervals of transition rates were obtained by using maximum likelihood (MLE) method and its related variance–covariance matrix. Transition probabilities can be derived from these instantaneous rates incorporating with age and sex. The likelihood ratio test was used for the model comparison between the current Markov model assuming that subjects in different severity of RMRC have different risks for CVD and the other one assuming that subjects in different severity of RMRC have equal risk for CVD with the likelihood ratio test. All analyses were conducted with the standard statistical software, SAS release 9.4 (SAS Institute Inc, Cary, NC).

## Results

At prevalent screens, 16,886 (35.55%) had none of individual MetS components, 22,480 (47.33%) had 1 or 2 abnormal individual components, and 8129 (17.12%) had MetS. Table 1 summarizes the baseline characteristics of the study participants according to FMD, MMD and MetS. The selected characteristics including age, sex, and individual comments of MetS at baseline in study cohort are summarized in Table 1. The prevalence of MMD and MetS were 47.33% and 17.12%, respectively. Males were more likely to have MetS. High blood pressure played a dominant role in the development of both MMD and MetS. Around half of subjects with MMD and 84% subjects with MetS had high blood pressure. 76% subject with MetS had abnormal central obesity. The transition history by RMRC at subsequent screens and the following vital status are also shown in Table 2. The results show that about 2.64% and 17.16% transited to MetS from FMD and MMD, respectively. Among those in FMD and MMD, around half remained in the same state. The proportions of occurrence of CVD among subjects of FMD, MMD, and MetS were 10.97%, 19.55%, and 29.30%, respectively. Around 0.69% CVD cases died from CVD during follow-up. The proportion of subjects dying from OCD in FMD, MMD, MetS, and CVD cases were 0.53%, 1.09%, 1.35%, and 1.86%, respectively.

**Table 1:** The selected baseline characteristics of the study participants by the status of MetS.

Characteristics	FMD	MMD	MetS
N (%)	16,886 (35.55)	22,480 (47.33)	8129 (17.12)
Age (years), mean ± SD	40.6 ± 11.8	47.8 ± 13.5	52.6 ± 13.0
**Age group, n (%)**			
20–29	2752 (16.30)	1764 (7.85)	259 (3.19)
30–39	5780 (34.23)	4863 (21.63)	1145 (14.09)
40–49	5020 (29.73)	6440 (28.65)	2033 (25.01)
50–59	2001 (11.85)	4480 (19.93)	2030 (24.97)
60–69	948 (5.61)	3265 (14.52)	1725 (21.22)
70 +	385 (2.28)	1668 (7.42)	937 (11.53)
**Sex, n (%)**			
Men	4296 (25.44)	9397 (41.80)	3679 (45.26)
Women	12,590 (74.56)	13,083 (58.20)	4450 (54.74)
Systolic blood Pressure (mmHg)	109.7 ± 10.9	127.5 ± 18.9	139.1 ± 19
Diastolic Blood Pressure (mmHg)	70.1 ± 7.4	80 ± 11.8	86.2 ± 13.2
Fasting glucose (mg/dl)	84.5 ± 6.8	92.5 ± 23.8	115.7 ± 48.4
Total cholesterol (mg/dl)	184.9 ± 33.5	195 ± 38.9	207.7 ± 42.1
Triglyceride (mg/dl)	74 ± 28.4	119.5 ± 88.8	230.2 ± 183.5
HDL cholesterol (mg/dl)	64.6 ± 12.4	56.6 ± 13.7	47.2 ± 12.3
**Criteria**			
Abnormal central Obesity, n (%)	–	6061 (26.96)	6206 (76.34)
Hypertriglyceridemia, n (%)	–	4812 (21.41)	5960 (73.32)
Low level of HDL, n (%)	–	4810 (21.40)	4332 (53.29)
High blood pressure, n (%)	–	12,190 (54.23)	6839 (84.13)
Impaired glucose Tolerance, n (%)	–	3650 (16.24)	4649 (57.19)

*FMD* free of MetS, *MMD* mild metabolic disorder with 1–2 criteria.

**Table 2:** Screening finding regarding the RMRC and occurrence of CVD and CVD death.

Transitions	Subject/screens	%
**At prevalence screening**		
Free of metabolic disorder (FMD)	16,886	35.55
Mild metabolic disorder (MMD)	22,480	47.33
Metabolic syndrome (MetS)	8129	17.12
**In the subsequent screening**		
*From FMD*		
Staying in FMD	4219	48.57
Progressing to MMD	3235	37.24
Progressing to MetS	229	2.64
Occurrence of CVD	953	10.97
CVD death	4	0.05
OCD	46	0.53
*From MMD*		
Regressing to FMD	1521	12.15
Staying in MMD	6252	49.94
Progressing to MetS	2148	17.16
Occurrence of CVD	2448	19.55
CVD death	14	0.11
OCD	137	1.09
*From MetS*		
Staying in MetS	3338	69.17
Occurrence of CVD	1414	29.30
CVD death	9	0.19
OCD	65	1.35
**From CVD cases**		
Censoring from CVD death or OCD death	4666	97.45
CVD death	33	0.69
OCD	89	1.86

Table 3 shows the estimated results of disease progression history for RMRC, the risk for CVD, and associated deaths from CVD and other causes. The estimated progression rate from FMD to MMD was 44.82% (95% CI 42.95–46.70%), whereas the regression rate was estimated as 29.11% (27.77–30.45%). The progression rate from MMD to MetS was estimated as 6.15% (95% CI 5.89–6.42%). The estimated annual incidence rates of CVD increased in proportion to number of criteria of MetS, being 1.62% (95% CI 1.46–1.79%), 4.74% (95% CI 4.52–4.96%), and 20.22% (95% CI; 19.52–20.92%) FMD, MMD, and MetS, respectively. The estimated hazard rate of CVD death was 6.1 (95% CI 4.6–7.7) per thousand. The corresponding figures of OCD increased with severity of metabolic disorder, from 1.1 (95% CI 0.8–1.4) per thousand for those FMD, to 9.1 (95% CI 7.2–10.9) among CVD cases. This model fitted better than the one assuming that subjects in different severity of RMRC had equal risk of developing CVD with the likelihood ratio test ($\chi_{\left( 2 \right)}^{2} = 4034.4, p < 0.0001$).

**Table 3:** Estimated results of disease progression history for RMRC, CVD and CVD death.

Parameters	Estimate (%)	95% CI (%)
Transition rate from FMD to MMD	44.82	42.95, 46.70
Regression rate from MMD to FMD	29.11	27.77, 30.45
Transition rate from MMD to MetS	6.15	5.89, 6.42
Incidence rate of CVD from FMD	1.62	1.46, 1.79
Incidence rate of CVD from MMD	4.74	4.52, 4.96
Incidence rate of CVD from MetS	20.22	19.52, 20.92
Hazard rare of CVD death	0.61	0.46, 0.77
Hazard rate of OCD from FMD	0.11	0.08, 0.14
Hazard rate of OCD from MMD	0.27	0.22, 0.31
Hazard rate of OCD from MetS	0.77	0.58, 0.95
Hazard rate of OCD from CVD	0.91	0.72, 1.09

Table 4 shows the estimated rate ratios of age and sex, adjusting for each other, on multi-state transitions associated with RMRC, CVD, and death from CVD or OCD. The results show that an increasing in one year of age would increase the risk of progression, such as from FMD to MMD, from MMD to MetS, and from MetS to CVD. However, age was negatively associated with the transition from MMD to FMD. An increasing year of age led to 8% (95% CI 5–11%) increased risk of CVD death, and 10% (95% CI 9–11%) risk of OCD death. Male had higher risk of progression rate (RR = 1.30, 95% CI 1.18–1.44) and lower risk of regression rate (RR = 0.67, 95% CI 0.61–0.75) from FMD to MMD than female. However, the incidence rates of developing CVD from free of MMD and MetS for male were lower than female. Nonetheless, male still had higher risk of dying from CVD (RR = 2.88, 95% CI 1.70–4.90) among CVD cases than female. So was the death rate of OCD (RR = 3.63, 95% CI 2.99–4.41).

**Table 4:** Effects of age and sex on multi-state progression associated with metabolic scoring status and CVD.

Transition rates	Age (one additional year of age)	Sex (male vs. female)
	RR (95% CI)	RR (95% CI)
Transition rate from FMD to MMD	1.01 (1.009, 1.017)	1.30 (1.18, 1.44)
Regression rate from MMD to FMD	0.97 (0.964, 0.973)	0.67 (0.61, 0.75)
Transition rate from MMD to MetS	1.03 (1.024, 1.031)	0.98 (0.90, 1.07)
Incidence rate of CVD from FMD	1.06 (1.049, 1.064)	0.50 (0.39, 0.66)
Incidence rate of CVD from MMD	1.04 (1.039, 1.046)	0.88 (0.81, 0.97)
Incidence rate of CVD from MetS	1.01 (1.01, 1.016)	0.86 (0.80, 0.92)
Hazard rate of CVD death, *λ*_45_	1.08 (1.05, 1.11)	2.88 (1.70, 4.90)
Hazard rate of OCD from FMD, MMD and MetS	1.10 (1.094, 1.111)	3.63 (2.99, 4.41)

**Figure 3:**
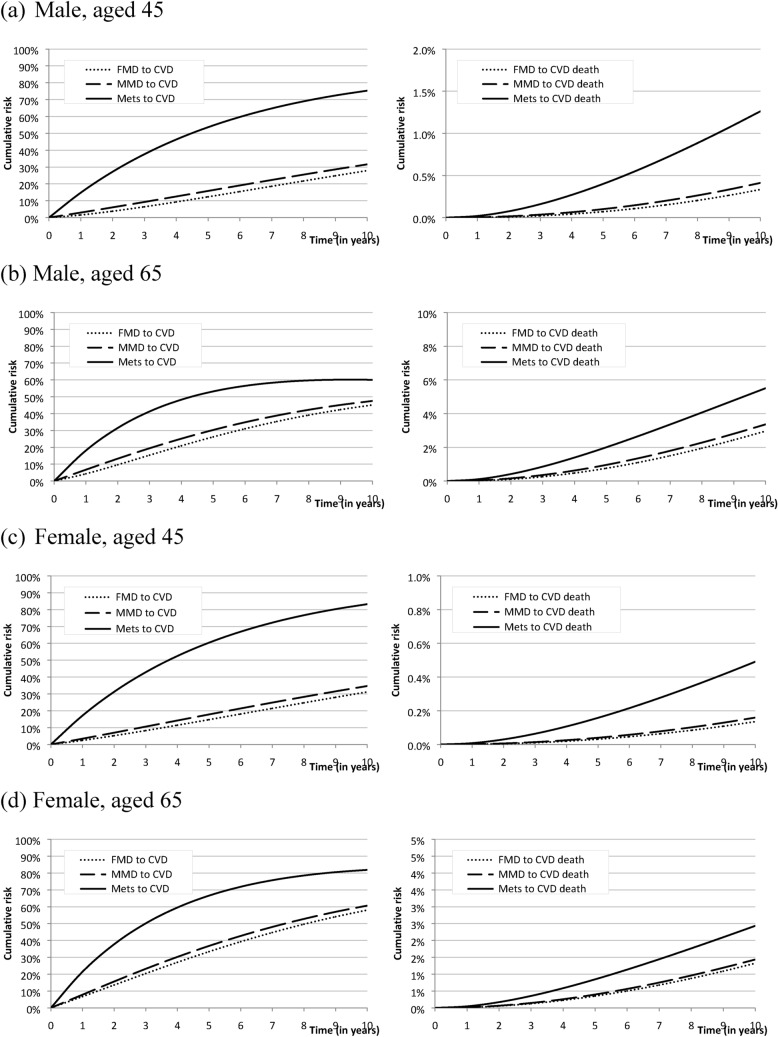
Cumulative risk of developing CVD and CVD death by different level of metabolic disorder for hypothetical male and female cases aged 45 and 65 years.

Figure 3a, b shows the cumulative risk of developing CVD and CVD death from FMD, MMD, and MetS of young (aged 45) and old (aged 65) male. Old male had higher probability of developing CVD from FMD and MMD but not MetS and also had higher proportion of dying from CVD compared with young male. The 10-year cumulative risks of dying from CVD among MetS for aged 45 and 65 were roughly 1.3% and 6.0%. The same trend was also noted in female (Fig. 3c, d). Compared with male, female had higher probability of developing CVD but lower risk of dying from CVD.

## Discussion

The Markov model which we used here to elucidate the dynamic change of MetS and CVD has been previously proposed to identify the metabolic factor initiating the progress of MetS with an evidence showing that dyslipidemia and obesity were more likely to initiate the dynamic progression of MetS^14,15^. In contrast to this previous study using a Markov model, our study focuses on the transitions between MetS-related status (FMD, MMD and MetS) in relation to the occurrence of CVD with allowance for the regression of MMD to healthy state. It is very interesting to note that approximately 30% MMD that were potential of being reversible to healthy state. Conventionally, one usually modelled the CVD incidence separately for three groups as healthy, MMD and MetS with Kaplan–Meier method and tested with log-rank test. However, such an independent comparison is not adequate for a linear progression form FMD, MMD, MetS until CVD allowing for the regression from MMD to FMD. The multi-state Markov model provide a solution. After taking into account regression, the risk for CVD increased with the severity of MetS being from less than 1.62%, 4.74%, and 20.22% annually. The wide gap of CVD incidence between MetS and FMD or MMD provides an indirect evidence to support the definition of MetS, any three or more of risk factors, rather than 1 or 2 factors and also indicates an obscure underlying pathophysiologic change when jumping into MetS state. Such a pathophysiologic change would further facilitate the progression of the disease and make the regression of MetS to previous state impossible without medical intervention.

The gradually increased hazards of total mortality other than CVD death and CVD with severity of metabolic disorder were compatible with previous study results^6,16^ revealed in Table 3. It was also very interesting to note that age was positively associated with all progression rates but negatively associated with the regression from MMD to FMD which indicated the regression was more likely to occur in young age and compatible with our previous similar studies related to chronical disease^17^. More importantly, the negative effect of age on regression rates provides an insight to delivery health promotion as early as possible for young age group. If additional covariates on different transitions can be modelled by the extension of the current Markov model the results would also enable one to consider personal clinical surveillance of subjects with metabolic syndrome in the future.

Considering the large burden of CVD worldwide, we urgently require tools to assess potential novel therapies. However, the long duration of clinical end-point trials required to establish an incremental benefit gives substantial restriction. In recent years, new biomarkers such as TNF-α, IL-6, IL-1, hs-CRP and cardiovascular imaging provide possible surrogate endpoints which might shorten the evaluation period. Incorporating these new biomarkers as adequate surrogate endpoints together with our current MetS-based multi-state Markov model would become a powerful tool for personalized risk assessment for CVD.

From previous study^18^, individual risk can be predicted and the application in clinical consultation is very useful. However, from population and policy-making viewpoint, it is still not enough yet. The missing part in the puzzle can be assembled by the results of this study. The current study outlines the dynamic change of the MetS and associated CVD which can be used in evaluating the impact of policy. For example, the changes of disease transition rates before and after intervention policies provide a rapid and unique method to judge the effect, not to mention about the great potency after combining with utility function for cost–benefit analysis.

There are some limitations in our study. First, as this is a large community-based screening a serious selection-bias issue related to socio-economic status and other heath behaviors is very unlikely. Moreover, the invitation to attend the screening is unrelated to MetS. The only concern is related to demographic features such as age and sex because in our study cohort, the subjects were not randomly selected but invited through the multiple-disease screening program given the existing nationwide screening program such as Pap smear screening. Therefore, those who attended in the early phase of this screening program were older age and greater proportion of women than the general population. The mean age of attendees was 48.7 (SD = 14.5) years compared with 38.5 (SD = 15.0) years in those who did not attend because the elder population were invited in priority to participate in the program at early phase. Another difference between the groups is the higher proportion of woman attendees (62.1%) compared with 45.5% in the underlying population. It may be that women were more likely to be willing to attend because Pap smear screening was declared as a nationwide screening program and cervical cancer was one of the leading female cancers in Taiwan. Although the selection-bias cannot be fully ruled out, we do think other factors except age and sex as affected by Pap smear screening are unlikely because this is a very large population-based screening program and the basis for invitations was completely unrelated to MetS status. Regarding the concern over age and sex, this accounts for why we have to provide age- and sex-specific findings when a six-state Markov process is used for modelling the transitions between metabolic disorders and their prognostic outcomes.

Second, the concern over the impact of medications and the influence on the classification of RMRC in our community-based study is addressed as follows. It is possible that MMD and MetS would be underestimated as some of subjects may receive medications and return to FMD when they had the uptake of screen. However, we think it may not have a substantial influence as the personal disease history including diabetes, hypertension, and hyperlipidemia (the majority) with treatment have been collected by questionnaire in each time of screen and has been considered as one of the RMRC based on five criteria following the definition of the joint scientific statement by Alberti et al.^10^ Therefore, the underestimated metabolic disorder due to medication effect may be less affected. More importantly, as it is a community-based study rather than a hospital-based study the majority of subjects before attending the KCIS has undetected RMRC and MetS if they do not have self-reported personal disease history on the detailed components of MetsS. Thus, the impact of medications on the dynamics of detailed components of RMRC is therefore limited.

Third, the treatment effect on dynamic change of MetS in the current study was not substantial and the impact of treatment on the prognosis of CVD and its death is one of crucial factors. Modelling the effect of treatment on two prognostic outcomes is not of main interest in the current study. The two solutions we proposed in this study to reduce the confounding effect of treatment on the first part of dynamics of natural course on the transitions between RMRC are described as follows. (1) The time-varying information of disease history has been considered for defining the metabolic status (FMD, MMD and MetS) in each time of screen as mentioned above. (2) Once the participants progress to MetS, we will regard them as MetS patients and retain as MetS during the follow-up even if the following data shows they regressed to FMD or MMD states due to treatment. However, the effect of treatment is not able to completely prevent, particularly for subjects with the severe status in some individual components of MetS. Fourth, the short follow-up time of CVD case and its death might underestimate the incidence of CVD and CVD mortality, especially in the young age group. Finally, modelling age and sex-specific findings is on the grounds of imbalance of age and sex distribution. Beyond these, more factors associated with the progression of MetS are worthwhile to investigate. However, modelling other risk factors like life style factors in multi-step process would be involved with a complex statistical technique which is beyond the scope of this study.

The Markov model is useful for describing the dynamic progress of MetS and development of CVD. The dynamic transitions of age and sex on the detailed components of MetS and subsequent progressions to CVD and its death were quantified. Male was associated with the development of MetS, whereas female was more likely to develop CVD. Regression from MMD to FMD was more likely to observe in female and young age group. These findings would be very useful either from precision medicine viewpoints for high-risk group identification or population viewpoints for the evaluation of intervention programs.

## Six-state Markov Model for RMRC associated with the risk for CVD and its death

A multistate Markov model is used to describe the development for longitudinal failure time data with a multistate process like our case of RMRC-CVD-Death. The important assumption of the Markov model is called the Markov property that given the state at time t the probability of any state after time t is independent of the history before time t. Therefore, the repeated transitions for the same subject can be regarded as independent observations as if they were from different subjects. In the study of natural course on RMRC associated with the risk for two prognostic outcomes, CVD and its death, making allowance for other causes of death, the model classifies patients into one of six distinct states including FMD, MMD, MetS, CVD, CVD death, and OCD at any given time point during the follow-up. A change among these states is called transitions and the transition times correspond to the times at which these changes occur. A six-state continuous-time Markov process (shown in Fig. 2) is used for delineating the state of an individual at time *t* lying within a state space Ω = {1, 2, …, 6}.

- 1 = free of metabolic disorder (FMD, free of metabolic factors).
- 2 = mild metabolic disorder (MMD, with 1–2 abnormal metabolic factors).
- 3 = metabolic syndrome (MetS).
- 4 = cardiovascular disease (CVD).
- 5 = death of cardiovascular disease.
- 6 = other causes of death (OCD).

Because we would like to model the natural history of RMRC, we assume that the regression from state 3 to state 2 was not allowed in the model. To model the instantaneous change of two state transitions between state 1 and state 6 as defined above, the intensity matrix Q is expressed as

where λ_12_ and λ_23_ represent the transition rate from state 1 to state 2 and from state 2 to state 3, λ_21_ represents regression rate from state 2 to state 1, and λ_*i*4_′s stand for the incidence rate of CVD from state *i* (1–3), λ_45_ is for hazard rate of CVD death, λ_*j*6_′s are specified for the death rate of OCD from state *j* (*j* = 1–4), respectively, taking other causes of death departing from the original states into account. Note that we here assume time-homogeneous Markov process in the current study. Namely, all λ’s are constant over time. The transition probability matrix, ***P***(*t*), which is the function of above transition matrix and time interval (*t*) for a specific transition, can be derived from the backward Kolmogorov equation, subject to ***P***(0) = ***I*** and by using the spectral analysis,

$${\varvec{P}}\left( t \right) = {\varvec{A}}^{ - 1} diag(e^{{d_{1} t}} , e^{{d_{2} t}} , \ldots ,e^{{d_{6} t}} ){\mathbf{A}}$$

where *d*_1_, *d*_2_, …, *d*_6_ are eigenvalues of intensity matrix (Q) and ***A***^−**1**^ and ***A*** are the right and left eigenvectors of ***Q*** matrix. The total likelihood function can be built up according to the observed transition modes.

In order to simultaneously model the effects of age and sex on the multi-state transitions between RMRC associated with the risk for two prognostic outcomes, CVD and its death, we applied the proportional hazard forms for the link between covariates and transition rates. The formula can be expressed as

$$\lambda_{ij} = \lambda_{ij0} \times exp\left( {\beta_{ij1} \times sex + \beta_{ij2} \times age} \right)$$

where the exponential transform of *β*_*ij*1_ and *β*_*ij*2_ represent rate ratios of sex (male for 1 and female for 0) and age (one additional year of age) for transition rate *λ*_*ij*_.

The maximum likelihood estimates (MLE) of the parameters of transition rates were obtained from the solution of the derivative of log-likelihood function. The variance–covariance matrix was derived from the inverse of negative Hessian matrix, evaluated at MLE. The asymptotic 95% confidence intervals (CIs) were obtained accordingly.

Statistical hypotheses for the two models tested in light of whether different severity of RMRC have different risk profiles of developing CVD, i.e.

$$\begin{aligned} H_{0} : \lambda_{14} & = \lambda_{24} = \lambda_{34} \\ H_{A} : \lambda_{i4} & \ne \lambda_{j4} \quad {\text{for}}\,{\text{any}}\,{\text{pair}}\,{\text{of }}\,i,j \quad (i,j = 1,2,3\quad {\text{and}}\quad ij) \\ \end{aligned}$$

The likelihood ratio test between models under H_0_ and H_A_ was used to test whether to suppor the null or alternative hypothesis.
